# Supplementary material for: Hybrid Models and Biological Model Reduction with PyDSTool
Source: PLoS Comput Biol. 2012 Aug 9;8(8):e1002628. doi: 10.1371/journal.pcbi.1002628 (PMC3415397; doi:10.1371/journal.pcbi.1002628)
Supplement: Text S4 — Complete source code for the PyDSTool package (version 0.88.120504). Includes API documentation and help files linking to web pages. This file is identical to the current public release on Sourceforge.net. (ZIP) [file pcbi.1002628.s004.zip › PyDSTool/html/toc-PyDSTool.Variable'-module.html]

xml version="1.0" encoding="ascii"?


Variable'


# Module Variable'

---

## Classes

HybridVariable  
 OutputFn  

VarDiagnostics

Variable  

## Functions

iscontinuous  
 isdiscrete  
 isinputcts  
 isinputdiscrete  
 isoutputcts  
 isoutputdiscrete  
 numeric\_to\_vars  
 pointset\_to\_vars  

## Variables

API

Continuous

Discrete

Inf

LargestInt32

NaN

\_1DimplicitSolveMethods

\_all\_complex

\_all\_float

\_all\_int

\_all\_numpy\_complex

\_all\_numpy\_float

\_all\_numpy\_int

\_complex\_types

\_float\_types

\_implicitSolveMethods

\_int\_types

\_num\_equivtype

\_num\_maxmin

\_num\_name2equivtypes

\_num\_name2type

\_num\_type2name

\_num\_types

\_pytypefromtype

\_real\_types

\_seq\_types

contained

isfinite

notcontained

null\_predicate

targetLangs

uncertain

---

[hide private]
